# One-Tube-Only Standardized Site-Directed Mutagenesis: An Alternative Approach to Generate Amino Acid Substitution Collections

**DOI:** 10.1371/journal.pone.0160972

**Published:** 2016-08-22

**Authors:** Janire Mingo, Asier Erramuzpe, Sandra Luna, Olaia Aurtenetxe, Laura Amo, Ibai Diez, Jan T. G. Schepens, Wiljan J. A. J. Hendriks, Jesús M. Cortés, Rafael Pulido

**Affiliations:** 1 Biomarkers in Cancer Unit, Biocruces Health Research Institute, Barakaldo, Spain; 2 Quantitative Biomedicine Unit, Biocruces Health Research Institute, Barakaldo, Spain; 3 Department of Cell Biology, Nijmegen Centre for Molecular Life Sciences, Radboud University Nijmegen Medical Centre, Nijmegen, The Netherlands; 4 IKERBASQUE, Basque Foundation for Science, Bilbao, Spain; University of Edinburgh, UNITED KINGDOM

## Abstract

Site-directed mutagenesis (SDM) is a powerful tool to create defined collections of protein variants for experimental and clinical purposes, but effectiveness is compromised when a large number of mutations is required. We present here a one-tube-only standardized SDM approach that generates comprehensive collections of amino acid substitution variants, including scanning- and single site-multiple mutations. The approach combines unified mutagenic primer design with the mixing of multiple distinct primer pairs and/or plasmid templates to increase the yield of a single inverse-PCR mutagenesis reaction. Also, a user-friendly program for automatic design of standardized primers for Ala-scanning mutagenesis is made available. Experimental results were compared with a modeling approach together with stochastic simulation data. For single site-multiple mutagenesis purposes and for simultaneous mutagenesis in different plasmid backgrounds, combination of primer sets and/or plasmid templates in a single reaction tube yielded the distinct mutations in a stochastic fashion. For scanning mutagenesis, we found that a combination of overlapping primer sets in a single PCR reaction allowed the yield of different individual mutations, although this yield did not necessarily follow a stochastic trend. Double mutants were generated when the overlap of primer pairs was below 60%. Our results illustrate that one-tube-only SDM effectively reduces the number of reactions required in large-scale mutagenesis strategies, facilitating the generation of comprehensive collections of protein variants suitable for functional analysis.

## Introduction

The assessment of gene nucleotide sequences with experimental and clinical purposes has improved with an exponential progress in the last decade. As a result, genome- and exome-wide DNA sequence data provide an immense pool of information on the molecular basis of life, with a wide scope of academic, biotechnological, and therapeutic applications [1–3]. However, an important gap exists between the high-throughput efficacy in collecting DNA sequence information from novel genes and gene variants, and the laborious possibilities to experimentally test the properties of the encoded proteins. In biomedicine, such is the case with the numerous somatic mutations identified in tumor samples from cancer patients, or in the germ-line of patients with hereditary disorders [4,5]. Thus, a comprehensive assessment of the contribution of the different residues from highly relevant proteins to their structural and functional properties remains a current demand in life science research and translational medicine. Although the need for specific high-throughput protein functional assays forms the subsequent major bottle-neck, a first step towards protein structure-function scanning is the generation of comprehensive collections of cDNA mutations cloned into appropriate expression plasmids, ideally facilitated by automated processes [6,7].

Randomized mutagenesis, by error-prone PCR or random-generated oligonucleotide libraries, has been extensively used to generate protein variants with a desired function [8,9]. This unbiased manner, however, fails in obtaining comprehensive yet defined collections of mutations. In its turn, site-saturation mutagenesis (*i*.*e*. substitution of an amino acid by each of the other 19 residues) is a suitable tool to create diversity and fine-tune biological activity of proteins [10,11], and it provides information on the amino acid constraints at defined positions within a protein [12]. PCR-based site-saturation mutagenesis relies on the use of primers with a random nucleotide sequence at the codon of interest. However, due to an unequal distribution of triplets in the genetic code and other methodological biases, the obtaining of all mutations is quite troublesome [7–9,13–16]. When naturally-occurring or disease-related mutations need to be studied, only a defined subset of amino acid substitutions per residue (single site-multiple mutagenesis) is required, favouring approaches to optimize the selective obtaining of mutated residues. Thus, many research questions beg for ways to efficiently obtain large collections of defined mutations.

One-step inverse PCR-based mutagenesis procedures, coupled to DpnI restriction enzyme digestion to remove the parental methylated and hemimethylated DNA, constitute the more widely used current methodology to perform site-directed mutagenesis (SDM) [17]. However, the methods rely on the design and use of rather long oligonucleotide primers that vary in length, which increases the cost of each mutagenesis and the possibility of primer-dimer or primer secondary structure complications. This has been improved in part by the development of alternative methods which use partially overlapping pairs of mutagenic primers, but still long primers are recommended and their design is rather complex [16,18–21]. Some other modifications of the DpnI-based SDM protocol include the use of three primers and a one-step gradient PCR [22], the running of two separate PCR reactions for each mutation [23], the enzymatic assembly of overlapping PCR products [24], or the running of two sequential PCR reactions using so-called megaprimers in addition to the mutagenic primers [25,26]. This may improve mutagenic efficiency but also increases costs and methodological complexity. Here, we present a simple one-tube SDM protocol that, by combining in a customized manner different templates and/or distinct mutagenic primer sets with fixed lengths, efficiently generates comprehensive collections of mutant cDNAs suitable for protein functional scanning.

## Materials and Methods

### Site-directed mutagenesis and bacteria transformation

Mutagenic primers of pre-defined length were designed with the mutated codon (minimal number of nucleotide changes) in the center of the primer, flanked by equal number of nucleotides (21+3+21 nucleotides in the case of 45-mer primers; 13+3+13 nucleotides in the case of 29-mer primers; mutated codon underlined). Features of mutagenic primers Q97H and D107A, designed by the web-based QuikChange Primer Design Program (www.agilent.com/genomics/qcpd), were as indicated in the corresponding table (QCM primers). Forward and reverse primers for each mutation were fully complementary. Q97H introduces an XbaI site and D107A eliminates a BglII site in the human PTEN cDNA sequence (NM_000314). For human PTPRZ-B, positions are based on the NM_001206838 entry. Plasmid templates (pRK5-PTEN, 6 kbp; pYES2-PTEN, 7.1 kbp; pENTR-PTPRZ-B, 7.3 kbp; pCDNA3-PTPRZ-B, 10 kbp) were isolated from the dam^+^ DH5α *E*. *coli* strain and dissolved in double-distilled water (ddH_2_O) at 5 ng/μl.

PCR was carried out in 25 μl of reaction mixture containing the following components: 14.25 μl ddH_2_O, 5 μl plasmid template (25 ng of total plasmid templates per reaction; when using combinations of templates, equal amounts of each plasmid were mixed to give 25 ng), 2.5 μl buffer 10X Pwo DNA polymerase (Roche), 1.25 μl dNTP mix (4 X 2.5 mM), 0.8 μM of mutagenic primers mix, and 0.1 μl Pwo DNA polymerase (5 U/μl; Roche). For individual mutagenesis, 1 μl of each mutagenic primer (at 10 μM in ddH_2_O; 2 complementary mutagenic primers per mutation) (Invitrogen) was used. For SDM using mixes of primer sets, the mutagenic primer pairs were premixed and 2 μl of the mix was used per reaction. PCR conditions were: 1 min at 95°C to denature the template, followed by 18 cycles of 50 sec at 95°C, 50 sec at 60°C, and 5 min at 68°C (7 min at 68°C for PTPRZ-B plasmids), and finally a 7 min extension at 68°C. PCR products were treated at 37°C during 2 h with 0.15 μl DpnI (20 U/μl; New England Biolabs) to digest methylated parental DNA, leaving the non-methylated amplified DNA containing the desired mutations. 2 μl of the DpnI-treated PCR product were mixed in a round-bottom 15 ml polypropylene tube with 10 μl TNE (50 mM Tris-HCl pH 7.5, 100 mM NaCl, 5 mM EDTA) and 10 μl TCM 10X (100 mM Tris-HCl pH 7.5, 100 mM CaCl_2_, 100 mM MgCl_2_) buffers, on ice. After 5 min (for cooling), 25 μl of competent DH5α *E*.*coli* (generated by the CaCl_2_ method [27]) were gently added and mixed, and kept 15 min on ice. Tubes were transferred to a 42°C water bath and incubated for 90 sec with occasional gentle shaking, followed by 5–10 min at 20°C. Subsequently, 0.5 ml of LB media was added to each tube and, following an 80 min incubation at 37°C under constant shaking, bacteria were transferred onto LB-Ampicillin plates and grown overnight at 37°C.

### DNA isolation, sequencing, and restriction analysis

Plasmid DNA was isolated from 3 ml of bacteria culture using a miniprep plasmid purification kit (NucleoSpin Plasmid EasyPure, Macherey-Nagel), and resuspended in 50 μl ddH_2_O. The yield of DNA obtained was about 0.5 μg/μl. Prior to sequencing, 3 μl of the purified plasmid DNA were digested with the appropriate restriction enzyme, resolved on 1% agarose gels, stained with Gel Red^TM^ Nucleic Acid Stain (Biotium), and visualized with UV light. These digestions allowed identification of the different templates when using plasmid combinations. About 300 ng of the purified plasmid DNA were processed for sequencing using the appropriate primers and an ABI 3130xl Genetic Analyzer (Life Technologies). DNA sequence analysis was done using BioEdit. In the case of the PTEN Q97H and D107A mutations, enzyme restriction analysis with XbaI or BglII, respectively, was performed to monitor the mutagenesis.

### Statistics, simulation, and programming

Previous algorithms have been created to estimate completeness and diversity in libraries generated by oligonucleotide-directed random mutagenesis [28,29]. In our standardized mutagenesis using mixes of defined oligonucleotides, we assume that random selections of individual mutations occur from the mutagenic mix. This procedure follows a multivariate hypergeometric probability distribution, which for a population with size *N* containing *c* different groups (mutations), models the probability of *k*_*i*_ successes (extractions of different groups; with *i* = 1, …, c) after *n* draws without replacement (each draw can be either a success or a failure [extraction of the same groups]), with the population having a total of *K*_*i*_ successes within each group. More precisely, for a population with size *N*, the probability of having *k*_*i*_ successes on a total *K*_*i*_ within *c* groups after *n* draws is given by:

$$Prob=\frac{\prod_{i=1}^{c}\left(\begin{array}{c} K_{i}\\ k_{i} \end{array}\right)}{\left(\begin{array}{c} N\\ n \end{array}\right)}$$ (1)

where the constraint $\sum_{i=1}^{c}k_{i}=n$ fulfills.

To calculate the probability of finding at least one mutation for each group from the population involves the calculation of all combinations not reaching the needed minimum to fulfill the goal. When *c* increases (see Eq 1), this calculation is computationally demanding, so calculations are shown up to a maximum of 6 different mutations). To overcome this limitation, we simulated the same process by randomly choosing individual mutations from the mutagenic mix. We developed a script running on Matlab (MathWorks Inc) which estimates probabilities by iterating 50000 times for every group (in good agreement with theoretical values).

A simple Python program (AlaChainRep) that lists standardized mutagenic primers for Alanine (Ala) replacement in a given chain is provided. In addition to standalone GUI executable files running on Windows and GNU/Linux, we also developed a simple functional version to work on-line. All details, source code and information can be found at http://erramuzpe.github.io/LAB_TOOLS

## Results and Discussion

### Effective one-step PCR-based site-directed mutagenesis using standardized mutagenic primers of pre-defined length

We performed a series of site-directed mutagenesis (SDM) experiments aimed to increase the yield and effectiveness of the mutagenesis process when large numbers of amino acid substitutions are wanted. These mutagenesis experiments were aimed at cDNAs encoding PTEN and PTPRZ-B, two proteins involved in human disease and of interest for comprehensive mutational analysis. Our mutagenesis procedure is based on the QuikChange^TM^ protocol (Agilent Technologies) and uses circular plasmids (containing the sequence to be mutated) as templates for an inverse PCR reaction, two complete-overlapping primers of pre-defined length containing the desired mutation, a high-fidelity DNA polymerase, and finally restriction enzyme DpnI. After DpnI-mediated elimination of the methylated template DNA, a fraction of the SDM reaction mix is directly used to transform *E*. *coli* bacteria.

**Fig 1:**
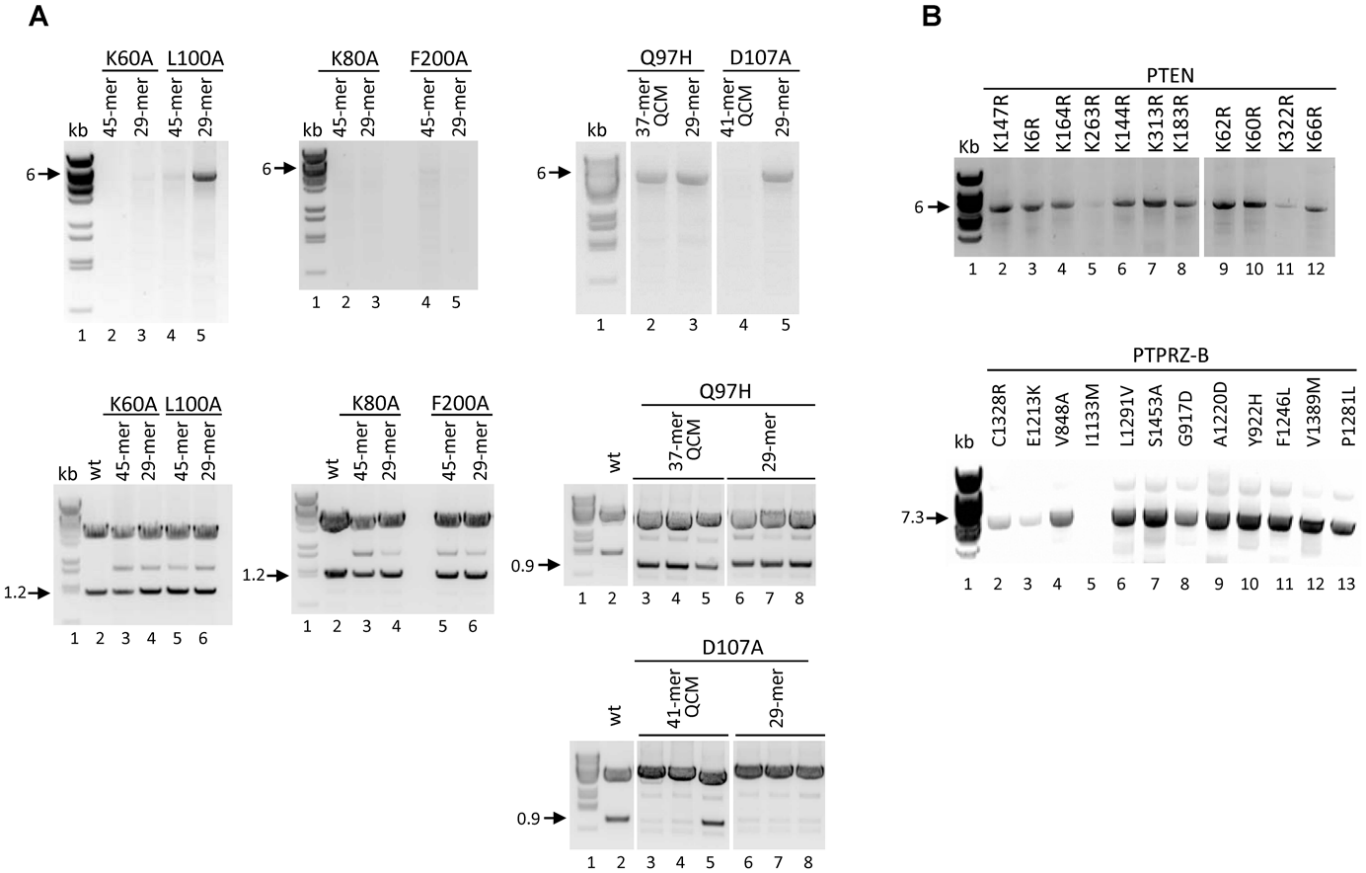
Amino acid substitution mutagenesis using mutagenic primers of different length, Tm, and GC composition. **(A)** Mutagenic primer pairs of pre-defined length (45-mer or 29-mer) or as suggested by the web-based QuikChange Primer Design Program (QCM primers) were used to create various PTEN mutations (pRK5-PTEN as template plasmid). Features of the mutagenic primers are listed in Table 1. Upper images show 10 μl of the respective PCR product, or of BstEII-digested λ phage as size marker (kb, lanes 1), following electrophoresis on 1% agarose gels. Lower panels display 1% agarose gel electrophoresis results for purified plasmid DNA (~3 μg) after restriction enzyme digestion. To reveal the 1.2 kbp PTEN insert (mutations K60, L100A, K80A, and F200A) an XbaI/SalI double digestion was used and one sample per SDM reaction is shown. To monitor the mutagenesis efficiency (mutations Q97H and D107A) we used XbaI/SalI or BglII, respectively, and three samples are shown per SDM reaction. Digested wild type pRK5-PTEN (wt, lanes 2) was included as a control. Gels correspond to experiments 1, 3, and 5 listed in Table 1. **(B)** PCR results using mutagenic (29-nucleotides) primer pairs generating 11 different Lys-to-Arg amino acid substitutions at different PTEN regions (upper panel; pRK5-PTEN as template plasmid), or generating 12 different amino acid substitutions at the intracellular region of PTPRZ-B (lower panel; pENTR-PTPRZ-B as template plasmid). Again, 10 μl of the PCR product was resolved on 1% agarose gels. Corresponding experiments and primer features are listed in Table 2. Molecular sizes of the linearized plasmids (6 and 7.3 kb) or the restriction enzyme-generated DNA fragments, are indicated left of the images in panels A and B.

An important constraint of current protocols is the frequent use of long oligonucleotide primers to achieve the recommended melting temperature (Tm) and GC content, which implies high costs and experimental complications when large numbers of mutations are required. In an attempt to optimize the SDM protocol for such purposes, we first performed comparative mutagenesis experiments using complete overlapping primers of different length, Tm, and GC content. Primers were designed to harbor the mutagenized codon in the center, flanked by oligonucleotide stretches of identical length at both sides (n+3+n, where n is the number of nucleotides flanking the underlined mutated codon). Primers were used at a final concentration of 0.4 μM (0.8 μM of primer pair per SDM reaction). We tested Pfu (Agilent Technologies), Pfx (Invitrogen), Phusion (Thermo Fisher Scientific) and Pwo (Roche) high fidelity DNA polymerases in preliminary experiments, and obtained comparable amplification results (not shown). All subsequent experiments reported here were performed with Pwo DNA polymerase. Different amino acids along the entire PTEN sequence, or along the intracellular region of PTPRZ-B, were targeted using our custom designed primer pairs and, as a comparison, we also used mutagenic primers resulting from the web-based QuikChange Primer Design Program (QCM primers; www.agilent.com/genomics/qcpd) (Tables 1 and 2). We found that reducing the primer length down to 29 nucleotides (13+3+13; which we now adhere to as standard mutagenic primer length), despite GC content and Tm alterations, did not substantially affect the mutagenesis efficiency as reflected by the amount of PCR product, the number of bacterial colonies obtained, or the mutation frequency (checked by DNA sequencing or restriction enzyme analysis). The amount of PCR product correlated with the number of bacterial transformants, but no relation was found between primer length, Tm, or GC content, and the amount of PCR product, the number of colonies after bacteria transformation, or the mutation frequency (Tables 1 and 2, Fig 1). In most cases, the amount of PCR product obtained suggested the yield of an exponential amplification reaction [30], whereas in some cases a small amount of PCR product was detected, although it was enough to give transformants with the desired mutation. The presence of a G or C at the primer ends did not affect mutagenesis efficiency.

**Table 1:** SDM using mutagenic primers of different length, GC content, and Tm.

		K60A^2^		L100A^2^			K80A^2^		F200A^2^			Q97H^1^		D107A^1^	
**Length**^a^		45*	29*	45	29		45	29*	45*	29		37* *(QCM)*	29*	41* *(QCM)*	29*
**%** **GC**		33	34	42	41		42	45	38	38		43	41	41	48
**Tm**^b^		76	65	80	68		80	70	78	67		78	71	79	74
**Colonies**^c^	*exp 1*	5	40	38	118	*exp 3*	3	25	17	5	*exp 5*	43	36	6	53
	*exp 2*	1	40	14	166	*exp 4*	2	16	1	10	*exp 6*	61	63	1	78
**Mutation frequency**^d^	*exp 1*	2/3	2/3	2/3	2/3	*exp 3*	3/3	1/3	3/3	2/3	*exp 5*	16/16	15/16	4/6	16/16
	*exp 2*	0/1	3/3	3/3	3/3	*exp 4*	2/3	2/3	1/1	3/3	*exp 6*	16/16	16/16	1/1	16/16

The superscripts in the distinct mutations indicate the number of nucleotide mismatches of each mutation with respect to the wild type sequence. pRK5-PTEN served as template plasmid.

*One of the primers from the mutagenic primer pair has G or C in 3’ position.

^a^Pre-defined length primers follow the design n+3+n (underlined is the mutated codon; 45-mer, 21+3+21; 29-mer, 13+3+13). QCM web-based primers were: Q97H, 13+3+16; D107A, 19+3+19.

^b^Tm was calculated according to the QuikChange^TM^ manual (Agilent Technologies).

^c^Number of bacteria colonies after transformation of the DpnI-digested PCR product; exp 1 to 6 are independent experiments.

^d^Number of colonies with mutation/number of colonies analyzed. Mutations were identified by DNA sequencing or, in the case of Q97H and D107A mutations, by DNA sequencing or restriction analysis.

**Table 2:** Standardized SDM using mutagenic primers of 29-mer length and different GC content and Tm.

***PTEN***		K147R*	K6R*	K164R*	K263R**	K144R*	K313R*	**K183R**	K62R*	K60R*	**K322R**	**K66R**	
**% GC**		52	48	45	41	41	38	38	34	31	28	24	
**Tm**		76	74	73	71	71	70	70	68.5	67	66	64.5	
**Colonies**	*exp 1*	59	73	80	7	70	86	63	111	87	25	71	
**Mutation frequency**	*exp 1*	3/3	3/3	2/3	2/3	3/3	3/3	3/3	3/3	3/3	1/3	3/3	
***PTPRZ-B***		**C1328R**	**E1213K**	V848A**	**I1133M**	L1291V**	S1453A*	**G917D**	A1220D*	**Y922H**	**F1246L**	V1389M*	**P1281L**
**% GC**		52	48	41	41	41	41	38	38	34	34	34	24
**Tm**		76	74	71	71	71	71	70	70	68.5	68.5	68.5	64.5
**Colonies**	*exp 2*	34	36	108	8	77	99	66	74	90	124	170	135
**Mutation frequency**	*exp 2*	3/3	2/3	1/3	0/3	2/3	3/3	2/3	3/3	3/3	3/3	3/3	3/3

All mutations have one nucleotide mismatch with respect to the wild type sequence. Data are represented as in Table 1. pRK5-PTEN or pENTR-PTPRZ-B served as template plasmids.

*One of the primers from the mutagenic primer pair has G or C in 3’ position.

**Both primers from the mutagenic primer pair have G or C in 3’ position.

Importantly, our 29-mer mutagenic primers were as efficient as the longer primers that were designed using the QCM web-tool, both in terms of PCR amplification and mutagenesis efficiency (Table 1, Fig 1). Routine experiments with the 29-mer primers generally resulted in 20–200 colonies per one-tenth of the SDM reaction product, of which about 85% contained the desired mutations. In a few cases (K80A, L100A, and F200A PTEN mutations) we also tested 25-mer primers, which gave yields comparable to the 29-mer primers. However, to avoid too low Tm values when using large numbers of different primers with the same length, we adhered to the 29-mer standardized primers. Primers that exceeded 40 nucleotides usually yielded lower numbers of colonies. Our finding that standardized 29-mer primers in the SDM protocol render mutations with high efficiency, and independent of their Tm, GC content or GC distribution, is in agreement with previous scanning mutagenesis analysis on Arrestin-1 and Gαi1 proteins [31]. Nevertheless, it may be that for difficult to amplify plasmid targets it remains necessary to further optimize mutagenic primer design and PCR conditions. We conclude that mutagenic primers of fixed pre-defined length provide efficient PCR amplification conditions and facilitate a high scale experimental setting for amino acid substitution SDM.

### One-tube-only SDM using combinations of cDNA plasmid templates

**Fig 2:**
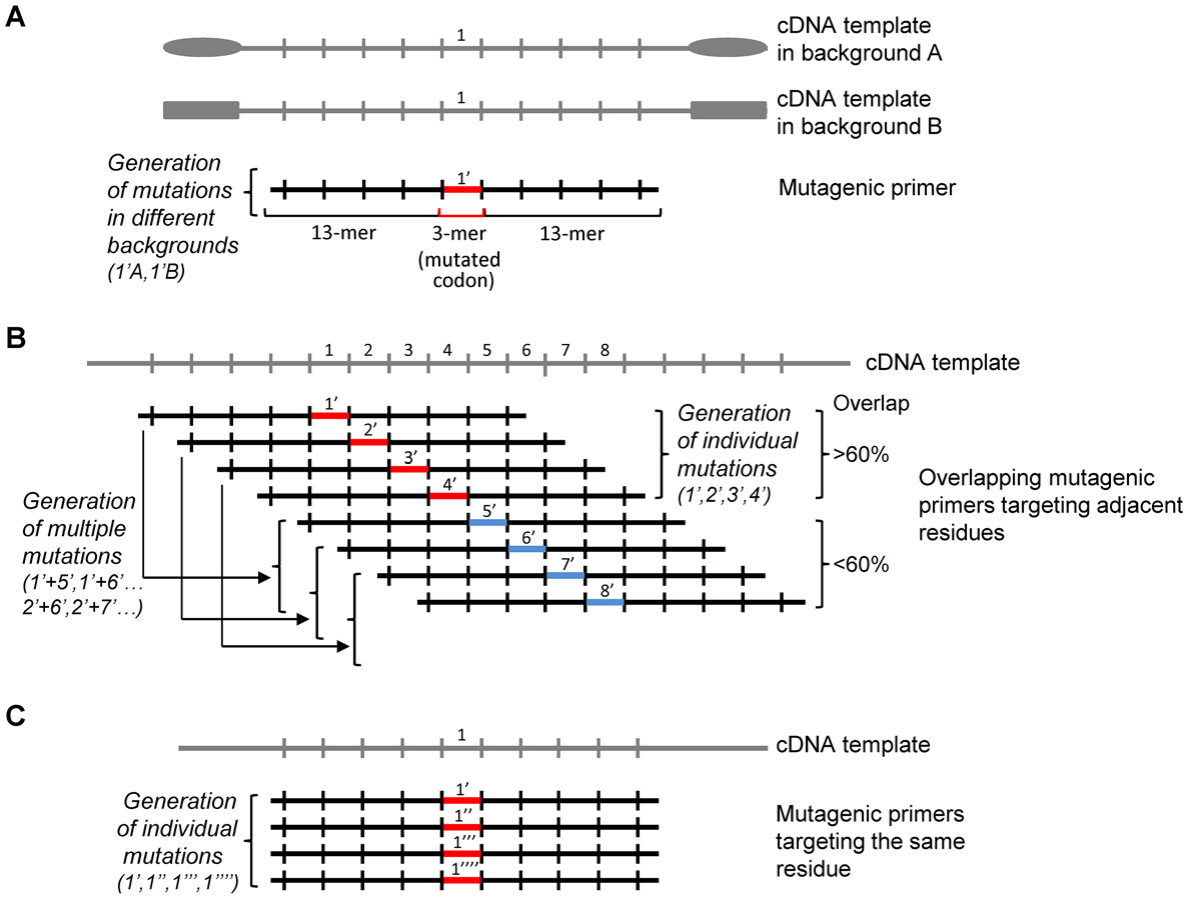
Schematic depiction of the different one-tube-only SDM approaches used in this study. The cDNAs are represented as lines divided in 3-mer base codons. **(A)** Strategy for the simultaneous introduction of a mutation (in red) in several background plasmids by a mixed templates SDM reaction. Wild type residue is indicated as 1, and mutated residue as 1’. The 13+3+13 design of our standardized mutagenic primer pairs is indicated. Plasmids are indicated as A and B. **(B)** Strategy for one-tube parallel substitution of sequential amino acids (scanning mutagenesis) by mixing overlapping mutagenic primers targeting adjacent residues in the SDM reaction. Wild type residues are indicated as 1 to 8, and mutated residues (in red and blue) as 1’ to 8’. Note that individual mutations are obtained when primer pair overlap is more than five codons, whereas multiple mutations are obtained when the overlap is smaller. **(C)** Strategy for the simultaneous substitution of one residue to a collection of distinct residues (single site-multiple mutagenesis) by mixing distinct mutagenic primers targeting the same residue in the SDM reaction. Wild type residue is indicated as 1, and mutated residues (in red) as 1’ to 1”“.

Since mutations in cDNAs of interest may need to be introduced in several distinct expression plasmids, each tailored for specific experimental purposes, we tested whether combining of different templates sharing a common cDNA insert would enable a one-tube means to derive collections of mutations (Fig 2A). We used plasmids pRK5-PTEN and pYES2-PTEN as templates, either alone or in combination, and different 29-mer (13+3+13) oligonucleotides as mutagenic primers (Table 3). PCR and mutagenesis efficiencies were not affected by the combination of templates, and mutants in both plasmid backbones were readily obtained, usually with a stochastic distribution. pRK5 and pYES2 vectors are similarly sized; 4.8 and 5.9 kbp, respectively. Since full-length PCR amplification of plasmids is dependent on their size, combinations of more differently sized plasmids as templates in SDM reactions may turn out less fruitful. Irrespective, combinations of similarly sized templates is a convenient approach to obtain cDNA mutations in multiple different plasmid backgrounds.

**Table 3:** One-tube-only SDM using combinations of template plasmids.

Template plasmid	Mutation targeted	Colonies	Colonies analyzed	Plasmids obtained	Mutation frequency
pRK5	N63A^2^	61	2	pRK5 (2/2)	0/2
pYES2	N63A^2^	95	2	pYES2 (2/2)	2/2
pRK5+pYES2	N63A^2^	58	4	pRK5 (2/4)	2/2
				pYES2 (2/4)	2/2
pRK5	N69A^2^	21	2	pRK5 (2/2)	2/2
pYES2	N69A^2^	5	2	pYES2 (2/2)	1/2
pRK5+pYES2	N69A^2^	35	4	pRK5 (2/4)	2/2
				pYES2 (2/4)	2/2
pRK5+pYES2	D22E^1^	8	4	pRK5 (2/4)	2/2
				pYES2 (2/4)	2/2
pRK5+pYES2	Y27C^1^	16	4	pRK5 (2/4)	2/2
				pYES2 (2/4)	2/2
pRK5+pYES2	P38H^1^	13	4	pRK5 (2/4)	2/2
				pYES2 (2/4)	2/2
pRK5+pYES2	Y88H^1^	61	4	pRK5 (2/4)	1/1
				pYES2 (2/4)	1/1
pRK5+pYES2	C105Y^1^	110	4	pRK5 (3/4)	1/1
				pYES2 (1/4)	1/1
pRK5+pYES2	R173C^1^	70	4	pRK5 (3/4)	1/1
				pYES2 (1/4)	1/1

The superscripts in the distinct mutations indicate the number of nucleotide mismatches of each mutation with respect to the wild type sequence. Data are represented as in Table 1. pRK5-PTEN and pYES2-PTEN, alone or in combination, served as template plasmids.

### One-tube-only SDM using combinations of standardized mutagenic primers targeting consecutive amino acids: implications in scanning mutagenesis

A high-resolution approach to study protein function is alanine scanning mutagenesis, in which the protein residues of interest are individually substituted for Ala, the amino acid with an inert non-bulky side chain [32]. Coupled to functional or biophysical assays, this allows to investigate structure-function relationships and to assess the contribution of each individual amino acid position to the functionality of the entire polypeptide. Unfortunately, scanning mutagenesis requires the design and use of a large number of primer pairs, hence is laborious, time-consuming and error-prone. Bioinformatics tools have been created to facilitate the large scale mutagenic primer design [31,33] but it remains cumbersome when large parts of a given protein need to be mutagenized. To help in the design of our fixed-length mutagenic primers for Ala scanning mutagenesis strategies, we compiled a user-friendly program called AlaChainRep. Rather than providing primers of different length or different relative localization of the mutated codon, our program renders a list of homogeneous primers with the same pre-defined length and following our n+3+n rule. The user defines the starting position of the first primer in the cDNA sequence and the primer length, the program then creates a list of primer pairs (forward and reverse) covering the remaining cDNA sequence and containing the desired Ala codon (with minimal nucleotide changes) in the center of each primer. Program and code sources are available at http://erramuzpe.github.io/LAB_TOOLS/. Although other programs exist that create amino acid-scanning mutagenic primers [31,33], our platform has the advantage of its easy implementation and simplicity, creating rapidly a useful list of mutagenic primers of fixed length.

**Fig 3:**
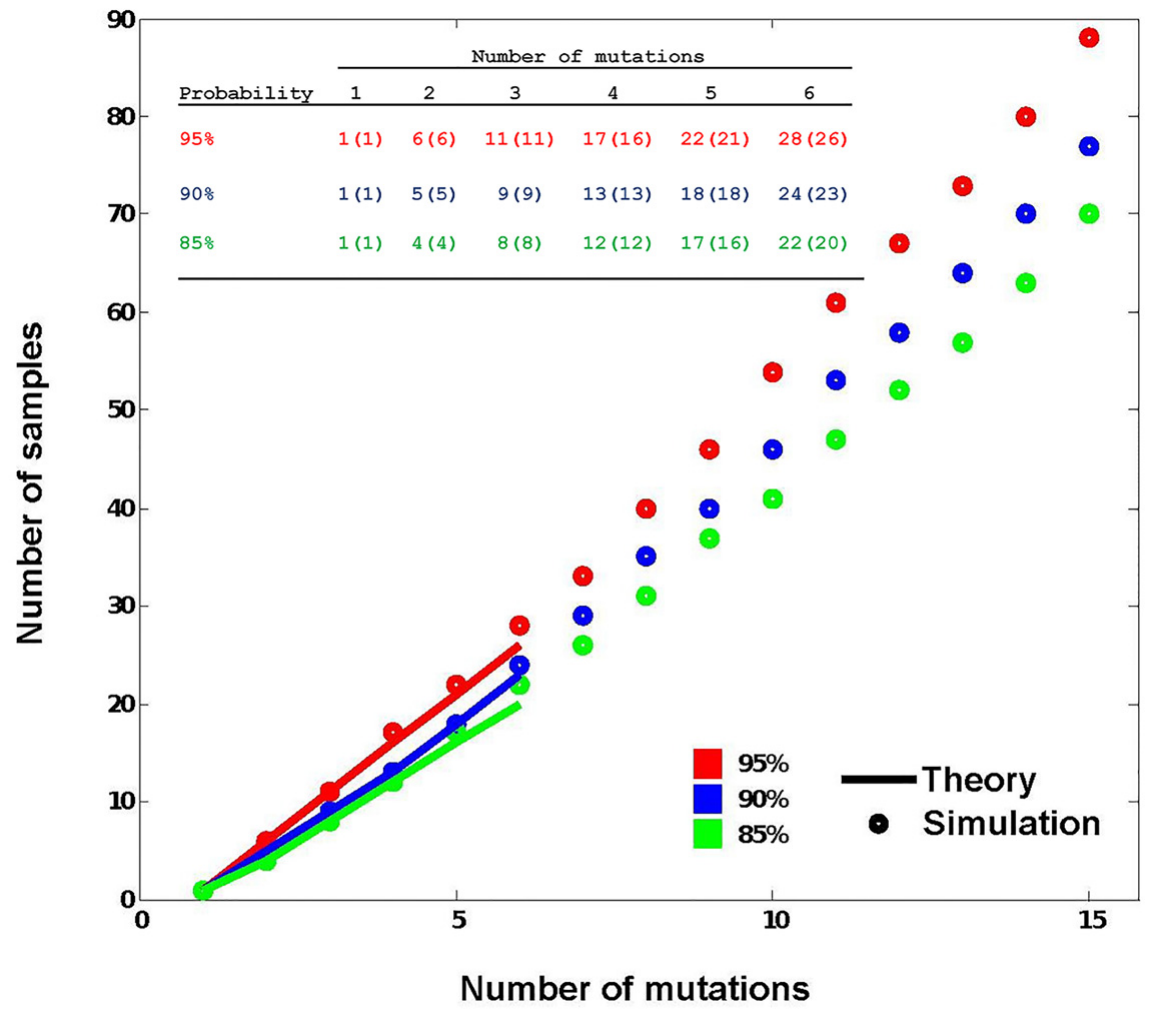
Data plots representing the obtaining (extraction) of all different individual mutations (samples to be extracted) from a given mix of mutations, assuming a stochastic distribution. The *x* axis indicates the number of different mutations included in the one-tube-only mutagenesis reaction, and the *y* axis indicates the number of samples (bacteria colonies) to be analyzed to obtain at least one of each mutation with a certain probability (95%, in red; 90%, in blue; 85%, in green). Dots indicate data obtained from a computer-assisted simulation, up to a mix of 15 different mutations. Solid lines indicate the probability distribution plots (Theory) up to a mix of 6 different mutations. Numeric values (non-brackets, simulation; brackets, theory) are indicated in the inserted table up to a mix of 6 different mutations.

In an attempt to maximize the efficacy of scanning mutagenesis, we performed SDM reactions using different combinations (2, 3, 4, 6, or 8) of mutagenic primer pairs targeting consecutive amino acids in the PTEN protein. Primers represent 29-mers as provided by AlaChainRep, and pairs are shifted three nucleotides relative to each other in the PTEN cDNA sequence (Fig 2B; Table 4). Primers in the mixture were used at equimolar concentrations to give a collective final concentration of 0.8 μM in the PCR reaction. Several approaches have been made to estimate diversity and completeness in libraries made by randomized SDM using degenerate primers [28,29,34]. To evaluate whether the obtaining of mutants distributes stochastically in our approach using defined mutagenic primers, we simulated a random selection of the individual mutations from the mutagenic mix (Fig 3). The obtained plots give an estimation of the randomness of obtaining the different mutations with respect to the number of different primer pairs used. For instance, combining four different primer pairs in the SDM reaction and assuming a mutagenesis efficiency of 100%, at least 13 resulting colonies need to be analyzed to obtain the four desired mutations with 90% probability. In general, the number of colonies we obtained after transformation with the PCR mutagenic product was not dependent on the number of combined mutagenic primer pairs, corroborating that it is the combined primer pair concentration that determines the yield of the PCR. However, the yield of the various individual mutations did not seem to follow a stochastic trend, as indicated by the frequent lack of obtaining all possible mutations for each combination of primer pairs (23% success rate [percentage of experiments in which all mutations are obtained; Table 4] versus an expected 88% [Fig 3]). Nevertheless, using from 2 to 4 consecutive primer pairs, it was possible to obtain all individual mutations in some instances (Table 4). Importantly, when the overlap between the different primer pairs used in the combined reaction was less than 60% (*i*.*e*. a distance of five codons in the targeted residue), also double-mutants were generated in the same one-tube SDM reaction (Fig 2B; Table 4). The feasibility to mutagenize both single and multiple positions within a given cDNA in one experiment is an added value when combinatorial mutations in a short stretch of amino acids need to be analyzed [35,36]. In conclusion, to obtain individual scanning mutations using 29-mer mutagenic primers, combinations of up to 4 consecutive primer pairs can be used, although the obtaining of mutations does not necessarily follows a stochastic trend. Combinations of more than 4 consecutive mutagenic primer pairs allows the additional generation of multi-site mutations in nearby residues in a single SDM reaction.

**Table 4:** One-tube-only SDM using combinations of primers targeting consecutive residues.

Mutations targeted^a^	Colonies	Colonies analyzed	Mutations obtained^b^^,^^c^
I50^2^-D51^1^	63	4	0-3-1
V54^1^-R55^2^	30	4	4-0-0
D58^1^-S59^1^	38	4	1-1-2
K60^2^-H61^2^	39	4	3-1-0
H64^2^-Y65^2^	166	4	0-4-0
Y68^2^-N69^2^	120	4	4-0-0
I50^2^-D51^1^-D52^1^	41	10	3-7-0-0
K60^2^-H61^2^-K62^2^	86	10	8-0-0-2
N63^2^-H64^2^-Y65^2^	82	10	0-3-5-2
I50^2^-D51^1^-D52^1^-V53^1^	28	14	0-7-2-4-1
V54^1^-R55^2^-F56^2^-L57^2^	35	14	4-4-3-1-2
K60^2^-H61^2^-K62^2^-N63^2^	52	14	6-1-6-0-1
H64^2^-Y65^2^-K66^2^-I67^2^	32	14	2-4-3-2-3
I50^2^-D51^1^-D52^1^-V53^1^-V54^1^-R55^2^	19	16	0-0-1-1-0-0-0 I50A+V54A (2) I50A+R55A (12)
K60^2^-H61^2^-K62^2^-N63^2^-H64 -Y65^2^	98	16	3-1-4-0-0-0-2 K60A+Y65A (6)
I50^2^-D51^1^-D52^1^-V53^1^-V54^1^-R55^2^-F56^2^-L57^2^	62	16	0-0-0-0-0-0-0-0-3 I50A+R55A (1) D51A+L57A (9) D52A+L57A (3)
K60^2^-H61^2^-K62^2^-N63^2^-H64^2^-Y65^2^-K66^2^-I67^2^	33	16	0-0-0-0-0-2-0-0-3 K60A+Y65A (1) K60A+K66A (3) K60A+I67A (2) H61A+K66A (1) H61A+I67A (3) K62A+I67A (1)

Data are represented as in Table 1. pRK5-PTEN served as template plasmid.

^a^All mutations are Ala substitutions (I50A, etc), and the superscripts indicate the number of nucleotide mismatches of each mutation.

^b^Number of samples obtained for each mutation are underlined and indicated following the order of the mutations targeted, and last number (not underlined) corresponds to wild type (for instance, in first row: I50A, 0 samples; D51A, 3 samples; wild type, 1 sample).

^c^The double mutations obtained are indicated, and the number of samples in each case are in brackets (for instance, in 14^th^ row: double mutation I50A+V54A, 2 samples).

### One-tube-only SDM using combinations of standardized mutagenic primers targeting the same amino acid: implications for single site-multiple mutagenesis

The generation of multiple mutant variants for the same amino acid position in a given protein is a solid approach to perform structure- and sequence-function relationship studies of amino acid regions of special relevance. In addition, functional residues in proteins whose gain- or loss-of-function is causative of disease, such as oncoproteins or tumor suppressors, are frequently targeted for different mutations whose functional analysis deserves scrutiny. To optimize the yield of multiple mutations of single amino acids, we performed SDM reactions using combinations of 29-mer mutagenic primer pairs targeting the same codon position but introducing different mutations (Fig 2C). Examples are provided in Table 5 for 2 different positions in PTEN that were mutated into 4 different amino acids. We used combinations of 1 to 4 mutagenic primer pairs on two different plasmid templates (pRK5 and pYES2). PCR and mutagenesis efficiencies were not affected by the combination of different primer pairs, and were similar for pRK5 and pYES2 plasmids. Noteworthy, use of combinations of primer pairs targeting the same residue yielded a stochastic distribution of the obtained distinct mutations upon analysis of appropriate numbers of colonies (87% success [Table 5] versus an expected 88% [Fig 3]).

**Table 5:** One-tube-only SDM using combinations of primers targeting the same residue.

Mutations targeted^a^	Colonies		Colonies analyzed		Mutations obtained^b^	
	pRK5	pYES2	pRK5	pYES2	pRK5	pYES2
**H61**						
R	62	141	2	2	2–0	2–0
Y	102	60	2	2	2–0	2–0
L	57	89	2	2	2–0	2–0
P	56	111	2	2	2–0	2–0
R-Y	91	113	4	4	1-2-1	4-0-0
L-P	84	79	4	4	2-2-0	2-2-0
R-Y-L	76	117	10	10	5-2-3-0	2-5-3-0
R-Y-L-P	40	95	14	14	4-4-3-1-2	5-1-3-2-3
**L70**						
A	236	247	2	2	1–1	2–0
P	290	280	2	2	2–0	2–0
F	276	300	2	2	2–0	2–0
I	244	180	2	2	2–0	2–0
A-P	224	114	4	4	1-3-0	1-3-0
F-I	358	133	4	4	2-1-1	1-3-0
A-P-F	180	163	10	10	1-7-1-1	1-4-4-1
A-P-F-I	156	185	14	14	1-4-5-1-3	0-6-1-3-4

Data are represented as in Tables 1 and 4. pRK5-PTEN or pYES2-PTEN served separately as template plasmids.

^a^The mutations targeted are indicated with the amino acid one-letter code (for instance, in second row: R, mutation H61R). All mutations have one nucleotide mismatch with respect to the wild type sequence, with the exception of L70A (two nucleotides mismatch).

^b^Number of samples obtained for each mutation are underlined and indicated following the order of the mutations targeted, and last number (not underlined) corresponds to wild type.

We also evaluated the usefulness of our one-tube-only SDM method for single site-multiple mutagenesis, using combinations of 29-mer mutagenic primer pairs that targeted individual amino acids in PTEN or PTPRZ-B for more than 6 different substitutions (Table 6). Such an approach has been explored before as a possible alternative for the use of mixes of degenerate mutagenic primers [37,38] Note that for such complex combinations of primers, a large number of samples need to be analyzed to obtain all mutations with high confidence from a single PCR reaction (Fig 3), which could compromise the efficiency of the one-tube-only SDM method. This may be partially overcome by performing iterative mutagenic reactions [11,39]. Using our one-tube procedure, a second sequential run of the one-tube-only PCR reaction was enough to obtain a good proportion of the mutations amongst a small number of samples. For instance, 15 and 12 different substitutions of the PTEN residues Ala126 and Gly129, respectively, were obtained when analyzing 24 samples resulting from two sequential one-tube-only PCR mutagenic reactions. Likewise, two rounds of one-tube-only mutagenesis rendered 16 different substitutions of the PTPRZ-B Leu1454 residue upon analysis of just 27 bacterial colonies (Table 6). The mutational success in the individual PCR reactions was slightly higher for cases that involved a single mismatch, as reflected by the frequency of obtained mutations (13/18), as compared to mutations with two mismatches (21/40) or involving full-codon mismatch (8/17). Our approach thus facilitates the obtaining of large numbers of different mutations for single residue positions, by performing several rounds of one-tube-only mutagenesis using mixes of standardized pre-defined length mutagenic primers. This is particularly useful when defined sets of multiple substitutions are wanted, such as when making collections of disease-associated single-residue mutations. The combination of sets of mutagenic primers creating individual multiple mutations in consecutive residues is also a possibility to increase the yield of protein variants from a single PCR mutagenic reaction [40]. In summary, the combination of pre-defined mutagenic primer pairs targeting the same residue and iterative PCR reactions boosts and simplifies the production of a stochastic mutation collection.

**Table 6:** Iterative one-tube-only SDM using large combinations of primers targeting the same residue.

Mutations targeted^a^	Colonies	Colonies analyzed	Mutations obtained^b^
**PTEN A126 1**^**st**^			
C^2^-E^2^-F^3^-H^2^-I^2^-K^3^-L^2^-N^2^-P^1^-Q^3^ R^2^-T^1^-W^3^-Y^3^	194	14	0-0-0-4-0-1-0-1-0-1 1-2-1-1-2
**PTEN A126 2**^**nd**^			
D^1^-E^2^-F^3^-G^1^-I^2^-L^2^-M^3^-P^1^-S^1^-V^1^	106	10	1-0-1-2-1-1-0-0-2-1-1
**PTEN G129 1**^**st**^			
C^3^-F^3^-H^3^-I^2^-K^2^-L^2^-M^3^-N^3^-P^2^-Q^2^ S^1^-T^2^-W^2^-Y^3^	24	14	0-0-1-5-0-0-0-0-0-0 0-2-4-0-2
**PTEN G129 2**^**nd**^			
A^1^-D^2^-E^1^-F^3^-K^2^-L^2^-M^3^-N^3^-P^2^-Q^2^ R^1^-S^1^-V^1^-Y^3^	68	14	2-1-3-0-0-0-1-0-0-2 1-0-2-1-1
**PTPRZ-B L1454 1**^**st**^			
A^2^-C^2^-D^3^-E^2^-F^1^-G^2^-H^3^-I^1^-K^2^-M^2^ N^2^-P^2^-Q^2^-R^2^-S^1^-T^2^-V^1^-W^2^-Y^2^	264	20	1-0-1-0-3-0-2-0-1-0 0-1-3-1-2-1-1-1-1-1
**PTPRZ-B L1454 2**^**nd**^			
C^2^-E^2^-G^2^-I^1^-M^2^-N^2^	11	7	0-2-0-3-2-0-0

Data and mutations obtained are represented as in Table 5. pRK5-PTEN or pCDNA3-PTPRZ-B served as template plasmids.

^a^Two iterative PCR mutagenic reactions (1^st^ and 2^nd^) were sequentially run for each targeted residue (A126, G129, and L1454). The mutations targeted in each case are indicated with the amino acid one-letter code, and the superscripts indicate the number of nucleotide mismatches of each mutation with respect to the wild type sequence.

^b^Number of samples obtained for each mutation are underlined and indicated following the order of the mutations targeted, and last number (not underlined) corresponds to wild type.

## Conclusions

Automation of protein functional analysis is a necessity when studying a high number of protein variants, as is the case for functional protein scanning of proteins with biotechnological or medical interest. Prior to any functional protein scanning, the optimization of the experimental conditions to create large numbers of defined mutations should be done. For such purpose, straightforward design and use of mutagenic primers, as well as a minimal number of PCR tube-reactions and bacterial transformations, are desirable. Here, we combined simple tools for mutagenic primer design and mixes of templates and/or primer pairs in iterative, single tube SDM reactions to rapidly and effectively obtain large numbers of defined amino acid substitutions in proteins, as a first step towards functional protein scanning methodologies. The design of our one-tube-only SDM (with regard to number and type of combinations of mutagenic primers and templates, and the number of sequential PCR reactions) allows further adaptation to custom experimental requirements, to provide maximal yield with minimal cost and time-effort. The systematic and iterative use of this standardized one-step inverse PCR-based SDM, aided by automatic design of pre-defined length mutagenic primers, will facilitate future comprehensive analyses of protein function.
